# A cross-cultural analysis on the Interpersonal meaning of attitude resources in Chinese and English online consumer reviews

**DOI:** 10.3389/fpsyg.2022.1001192

**Published:** 2022-10-05

**Authors:** Weichao Wang, Xiaofen Zhang

**Affiliations:** School of English for International Business, Guangdong University of Foreign Studies, Guangzhou, China

**Keywords:** attitude, online consumer review, interpersonal meaning, national culture, Chinese and English

## Abstract

Online consumer reviews benefit not only buyers but also sellers in the virtual market place. For consumers, they can realize their attitudes to products through the various lexical attitudinal resources indicating emotion and judgment, and for sellers they act as a form of customer feedback which can enhance the relationship between buyers and sellers. In that sense they improve the operation of the market price mechanism. The purpose of this study is to investigate the Interpersonal meanings realized through the attitude resources drawn upon by English and Chinese online consumers. Based on the Appraisal System, especially the Attitude System in Systemic Functional Linguistics (SFL), this study conducts a contrastive analysis of the attitudinal resources employed by consumers in Amazon UK and Amazon China. These features are analyzed by using the UAM CorpusTool to annotate the relevant resources, with similarities and differences in the general distribution of attitudinal resources identified, and any potential underlying reasons explained. The results of this study suggest that different features and distribution of attitudinal resources are employed in English and Chinese online consumer reviews, and that more attitudinal units are involved in English online consumer reviews than in the Chinese versions. Consumers from the United Kingdom also seem to use more affect resources than Chinese consumers in their online reviews, while Chinese consumers employ slightly more judgment resources and more appreciation resources. Possible factors that may cause such differences are examined in terms of the differing contexts of cultures.

## Introduction

More than half of US adults regularly read online consumer reviews before making a purchase (Pew Research Center, 2016), and 81% of 18–34 year-olds seek online consumer reviews before purchasing a product or service. Moreover, since the entry of Kindle into China market in 2013, it quickly occupied a large share of the domestic digital reading market; by the end of 2016, China has become the number one market for Amazon Kindle sales; during 2013–2018, the sales volume of Kindle in China reached millions of units, and the total number of books in Kindle China e-Bookstore in 2018 was nearly 700,000, to a large extent, Kindle influenced China’s digital reading market. According to China Digital Reading Report 2021, the number of digital reading users reached 506 million, with a growth rate of 2.43%, and the number of e-readers *per capita* was 11.58; while in the United Kingdom, since the beginning of 2012, an average of 114 e-books have been downloaded by consumers for every 100 paperback books sold by Amazon. The launch of series of Kindle devices has also stimulated readers’ desire to read. Consumers are now buying four times as many books as they did before they owned a Kindle. Amazon has now sold over 500,000 Kindle e-books in the United Kingdom.

Online consumer reviews, also called electronic word-of-mouth, have increasingly become a popular mode of Internet-mediated communication (Vásquez, 2014). As a type of user-generated content (UGC), online consumer reviews are comments given by consumers who have already experienced the interactional activity of purchasing certain goods or services online. Before making purchasing choices, potential consumers often refer to related reviews published on the relevant website. They draw on the fact that UGC provides a free consultancy for consumers before they make a purchasing decision (Feng and Ren, 2020).

With such a significant role Amazon plays in the e-reading market and the importance of online consumer reviews, it is imperative to delve into the consumer review discourse from linguistic perspective to investigate and compare the implied and expressed attitudes, and to uncover the possible underlying cultural factors.

The research literature on online consumer reviews has mainly focused on their economic value (Gong et al., 2012; Li and Wang, 2015; Wei and Zheng, 2018), their utility (Mudambi and Schuff, 2010; Skalicky, 2013; Filieri, 2015), the effects of cultural background (Kim et al., 2018; Kim, 2019), and more recently, a few other papers discussing the role of culture in exploring the determinants of the “perceived helpfulness” of online consumer reviews (Biswas et al., 2021; Filieri and Mariani, 2021). Other studies have investigated various linguistic features such as rhetorical moves, impoliteness, linguistic adaptation and mitigation involved in online reviews (Virtanen, 2017; Ren, 2018; Cenni and Goethals, 2020; Feng and Ren, 2020; Ho, 2020).

However, online consumer reviews are rarely investigated for their evaluative language, especially context-dependent evaluation (Pounds, 2011). Online consumer reviews are written to make comments on previous purchasing experiences, in which personal attitude will definitely be involved. Personal attitude expressed or implied in online reviews provide positive or negative information to potential consumers, which can thereby greatly influence their purchasing decisions (Ghose and Ipeirotis, 2010), and then the sales and reputation of the product (Wei and Zheng, 2018).

Similarly, the existing linguistics research on online consumer reviews lacks deeper investigations into the similarities and differences in attitudinal expressions between different national cultures, although cross-cultural analyses on the cultural effects of online consumer reviews from the perspective of review helpfulness have already be conducted by several scholars (Kim et al., 2018; Kim, 2019; Srivastava and Kalro, 2019; Jia, 2020). A contrastive analysis on the attitude resources employed in online reviews in different languages will not only improve the understanding of how consumers from different cultures express their purchasing experience evaluations in a clearer way, but also help to identify the needs of different consumers from different backgrounds (Zhu et al., 2019b).

## Literature review

This study aims to investigate the Interpersonal meaning of attitude resources in Chinese and English online consumer reviews, and to clarify the similarities and differences between attitude resources employed and the Interpersonal meaning realized in Chinese and English online reviews. The following literature review discusses the characteristics of the existing research over three areas: online consumer reviews in general, the resources utilized for expressing attitude, and the constituents of Interpersonal meaning in the Systemic Functional Linguistics (SFL) model.

### Online consumer reviews

Online consumer reviews are statements made by consumers about a specific product or service, which are published online and therefore are considered as a typical kind of electronic word-of-mouth (Filieri, 2015). Providing various product or service information, online consumer reviews play an important role in influencing consumers’ purchasing intentions or choices (Lee and Lee, 2009; Gupta and Harris, 2010) and published online consumer reviews may help potential consumers to find products or services that might meet their needs and preferences (Dellarocas, 2003). Analyzing online consumer reviews helps marketers to better understand the needs of consumers and may accordingly improve related offerings or services (Laroche, 2010).

Previous studies on online consumer reviews have recognized the significance of online consumer reviews. On one hand, the economic value of online consumer reviews has been primarily explored from a more seller-oriented perspective. The volume and valence of online consumer reviews have been found to positively affect product sales and thus brand loyalty (Dellarocas et al., 2007; Gong et al., 2012; Li and Wang, 2015; Wei and Zheng, 2018). From a more consumer-oriented perspective however, the impact of existing reviews on prospective consumers, or the helpfulness of online consumer reviews, has also been examined. Online reviews providing certain information, such as comprehensive content (Srivastava and Kalro, 2019) and experience-based information (Skalicky, 2013; Filieri, 2015), are revealed to be more helpful to potential consumers.

The above studies, in establishing the importance of online consumer reviews to both sellers and prospective consumers, pay little attention however to online review writers who create value for companies without the need to pay anyone wages (Ritzer and Jurgenson, 2010). The primary motivations for consumers to post online reviews include concern for other consumers, desire for social interaction and desire for economic incentives (Hennig-Thurau et al., 2004). Some scholars have recently attempted to comprehend online review writers from linguistic perspectives. Those studies show that specific linguistic strategies are used by review writers in online consumer reviews to achieve certain purposes, such as projecting reliability (Virtanen, 2017), constructing home or domestic feeling (Zhu et al., 2019a) and presenting impoliteness (Feng and Ren, 2020). One of the most prominent reasons for review writing is self-expression (Vásquez, 2014), while some computational studies provide insights to evaluations and opinions of online review writers through sentiment analysis, classifying positive and negative emotions or feelings in published consumer reviews (Guerreiro and Rita, 2020; Xu, 2020).

However, few studies intend to explore evaluations in online consumer reviews through more detailed discourse analysis. The use of automated sentiment analysis is limited for identifying evaluations in online consumer reviews, as evaluation is context-specific (Pounds, 2011; Vásquez, 2014), and because a word classified as positive in one sentence can be negative in another. Moreover, some evaluation is implicitly conveyed by review writers, which is quite difficult for computer tools to identify (Vásquez, 2014). One of the limited discourse studies that can be referred to is that of Tian (2013) who applies Engagement System, one of the three subsystems of Appraisal System (Martin and White, 2005), to explore travelers’ engagement patterns in their online hotel reviews. However, the Engagement System only deals with “sourcing attitudes and the play of voices around opinions” (Martin and White, 2005, p. 35). Similarly, in the present study, we will also examine the attitudes and opinions in online reviews through discourse analysis. Rather than focusing on the source of attitudes, the present study will however focus more on review writers’ attitudes *per se*. Online reviews are replete with evaluations which reflect “the speaker’s or writer’s attitude or stance toward, a viewpoint on, or feelings about the entities or propositions that he or she is talking about” (Hunston and Thompson, 2000, p. 5). Therefore, detailed analyses of attitudes are necessary to clarify how products are evaluated in online reviews, especially in terms of any contrastive cultural features between English and Chinese consumers. Accordingly, this study will concentrate on exploring specific and detailed attitudes expressed in online consumer reviews texts, from both English and Chinese consumer perspectives.

### Attitude resources

Attitude system is a subsystem of Appraisal system within the framework of SFL (Halliday and Kirkwood, 1978). There are three meta-functions in SFL, namely ideational function, textual function and interpersonal function, among which interpersonal function can be realized by linguistic manifestations of stance—people’s attitudes about or commitments to a person or proposition (Biber et al., 1999). And appraisal system provides a tool for researchers to closely analyze these linguistically manifested stances by uncovering meaning across whole texts (Martin and Rose, 2003). Attitude system, as a subsystem of appraisal system, is concerned with how attitudes are encoded in a text. Attitude system involves three types of attitude resources, which are affect, judgment and appreciation. Meanwhile, attitude resources are lexical units in oral or written evaluations that express personal feelings, judgment or appreciation. Affect focuses on people’s expressing emotional reactions such as happiness, satisfaction, security and inclination, and the use of affect distinguishes anecdotes from narratives. Affect resources can be used in combination with graduation. For example, “a very sad day” is a graduated form of “a sad day” (affect: unhappiness). Judgment refers to speakers’ ethical evaluation of people, including social esteem (personal judgment of admiration and criticism) and social sanction (moral judgment of admiration and criticism). Appreciation shows speakers’ esthetic evaluations of things, phenomena, or processes, we can evaluate a thing in terms of our “reaction” to it, its “composition” and its “value” (Martin and White, 2005). As a major subsystem in the Appraisal System, the Attitude System contributes to analyzing how the speaker or writer evaluates people and things (e.g., Marshall et al., 2010; Cabrejas-Peñuelas and Díez-Prados, 2014). It is a useful tool for the discourse analysis of various genres such as tweets (Ross and Caldwell, 2020), personal emails (Ho, 2014), public statements (Meadows and Sayer, 2013), and doctors’ discourse (Gallardo and Ferrari, 2010).

Those attitudinal studies, however, mainly deal with evaluative texts in one single language, even though two or more cultural contexts are involved. For example, Ho (2014) analyzes the evaluative lexis in English e-mails exchanged between Chinese professionals in Hong Kong and finds that rapport could be managed by using evaluative language in the professional workplace request e-mail. According to Vásquez (2014), studies on languages other than English may promote the understanding of the linguistic features of online consumer reviews and the localization of the social practice. Following this view, Ren (2018) investigates Chinese online consumer reviews on Amazon China and finds that different types of mitigation devices are used in Chinese consumer reviews. This recent endeavor still deals with texts of one single language, however.

For international online shopping platforms such as Amazon, consumers from different countries are targeted, and their diverse needs can be identified through recognizing the differences between them and how they adopt different communication styles or strategies in their online reviews (Zhu et al., 2019b). However, the more specific differences between attitudinal expressions in online reviews across different languages is an area where there is a dearth of research.

Language use, “the continuous making of linguistic choices” (Verschueren, 1999, p. 55), can be influenced by various factors, among which cultural context is one of the most important. The cultural variations in the use of linguistic resources have already been greatly recognized in established studies (e.g., Yang, 2013; Zhu et al., 2019b; Cenni and Goethals, 2020; Feng and Ren, 2020); for example, more hedges and boosters have been found in the US corporation discourse compared to the Chinese (Lee, 2020). From the perspective of Hofstede’s cultural dimensions (Hofstede, 2001), such as individualism vs. collectivism, Kim et al. (2018) find that customers from Western countries tend to be more positive in their ratings. It is then important for cultural contexts to be considered in the case of multilingual texts, and there is a need to explore cultural factors when online consumer reviews of different languages are studied. Drawing upon this prior research, this study will examine the similarities and differences between the use of attitude resources in Chinese and English online consumer reviews.

### Interpersonal meaning

In Systemic Functional Linguistics (SFL), language is interpreted as being fundamentally used to make meanings, and the Ideational, Interpersonal and Textual Metafunctions are used to describe these general meanings, or purposes (Eggins, 2004; Halliday, 2014). With the other two kind of meanings focusing on how to present experience in language and how to organize information in language respectively, Interpersonal meaning is comparatively more related to the relationship between the writer or speaker and the reader and the attitude of the writer or speaker toward the subject discussed (Eggins, 2004).

Recognizing the fundamental purpose of language use, a range of researchers have contributed to analyzing Interpersonal meaning in various texts, some examples being advertisements (Xia, 2020), and online newspaper comments (Gómez González and Taboada, 2021). Various linguistic resources which realize different Interpersonal meanings in those texts have also been explored. For example, metadiscourse in Chinese WeChat public account advertisements has been found to be helpful for advertisers to build solidarity with readers and to manage corporate image (Xia, 2020). Through a corpus-based approach, reportative adverbs in English have been shown to present the information discussed as well as the speaker’s position on the information (Rozumko, 2019). The transitivity patterns of gender-related sentence examples in Chinese dictionary have also been found to construct different images of females and males and to reflect different gender ideologies (Hu et al., 2018). Findings from the above studies all show that in order to establish certain relationship or express certain attitude, different linguistic resources can be used, and their usage realizes Interpersonal meanings.

Interpersonal meaning in online consumer reviews however is not sufficiently examined. Seemingly unilateral statements of review writers, online consumer reviews actually play important role in expressing personal attitude as well as constructing writer-reader relationships (Vásquez, 2014). For example, Hennig-Thurau et al. (2004) summarized several motivations of consumers publishing online reviews, which include expressing positive and negative feelings from the purchasing experiences, providing advice for potential consumers, and even helping the company in product sales. Moreover, Interpersonal meaning can always be found in online consumer reviews which are abundant in the language of evaluation, as appraisal resources function to realize Interpersonal meaning in various contexts (Querol-Julián and Fortanet-Gomez, 2012; Smith and Adendorff, 2014). Therefore, recognizing the relationship between evaluation and Interpersonal meaning, this study endeavors to investigate this aspect in online consumer reviews.

### Cultural factors

Culture is the collective programming of the mind (Hofstede, 2001) or a set of understanding shared among persons who have been similarly socialized (Terpestra and David, 1991). Cultural factors are vital for the understanding of communication system of discourse.

As Hall (1976) points out, culture is communication and communication is culture. For specific analysis, Hofstede’s cultural dimensions (Hofstede, 2001) Collectivism *VS* Individualism and Indulgence *VS* Restraint and Hall’s (1976) high- and low-context cultures could offer useful lens. Collectivism is emphasizing the interest of group and nation; people have obligated to pay close attention to family, society, etc. And they rely on social relationship. Meanwhile, individualism is individual-centered that the interest of individual person is more important than collectivist interest. People from more indulgent societies tend to enjoy life and be more positive, while people from more restrained societies attach great importance to moral disciplines and tend to be more pessimistic (Hofstede et al., 2010). Meanwhile, in low-context cultures, information and meaning are explicitly stated and they value direct verbal interaction; while in high-context cultures, individual internalizes meaning and information so that less is explicitly stated and they tend to value indirect verbal interaction (Hall, 1976).

### Summary

In sum, this study aims to investigate the Interpersonal meaning of attitude resources or units in Chinese and English online consumer reviews. They will be annotated and interpreted using the *UAM CorpusTool 3.0 and* the Attitude System as defined in SFL (Martin and White, 2005). From a cross-cultural perspective, the similarities and differences between the distribution of attitudinal resources in Chinese and English online consumer reviews will be identified, and any implication to be drawn from the differing cultural contexts will be discussed.

This study therefore attempts to explore the following research questions:

1. What attitude resources are employed in Chinese and English online consumer reviews to realize Interpersonal meaning?
2. What are the similarities and differences in the employment of attitude resources between Chinese and English online consumer reviews to realize Interpersonal meaning?
3. What are the possible factors accounting for the similarities and differences in the employment of attitude resources between Chinese and English online consumer reviews to realize Interpersonal meaning?

## Materials and methods

This study employs three theoretical constructs, namely Attitude system (Martin and White, 2005) for the detailed discourse analysis of the attitude resources used, construction of interpersonal meaning (Eggins, 2004; Halliday, 2014) for the similarities and differences, and cultural dimensions of national culture (Hofstede, 2001; Hofstede et al., 2005) and high- and low-context cultures (Hall, 1976). As we argue in literature review, attitudinal study is a useful tool for the discourse analysis of various genres such as tweets (Ross and Caldwell, 2020), personal emails (Ho, 2014), public statements (Meadows and Sayer, 2013), and doctors’ discourse (Gallardo and Ferrari, 2010). The previous studies mainly deal with evaluative texts in one single language, even though two or more cultural contexts are involved. In this article, we will include both English and Chinese data for our analysis to unveil the similarities and differences, and then to uncover the underlying cultural factors. Since we are all born and raised in certain cultures, our cognition is shaped by a certain culture. In order to better investigate the discursive features that surfaced, it is imperative to employ Hofstede’s cultural dimensions (Hofstede, 2001) and Hall’s high- and low-context cultures (Hall, 1976) to unveil the underlying cultural factors that are working beneath the specific linguistic features.

The methods utilized in examining the Interpersonal meaning of attitude resources in Chinese and English online consumer reviews are as follows:

### Data collection

The research subject is online consumer reviews in Amazon UK^1^ and Amazon China.^2^ As an international platform with websites of different countries, Amazon provides an enormous amount of online consumer reviews in different languages which are easily accessible for researchers due to the fact that those online consumer reviews are openly published. Explorations of online consumer reviews in different languages and in different cultural contexts would contribute not only to further understanding about different languages and cultures, but also raising an awareness the different aspects of consumer needs in different countries.

As presented in Table 1, a total of 300 consumer reviews including 150 sample texts from Amazon UK and 150 sample texts from Amazon China are collected. Among the 150 English reviews, 75 are marked as “positive” reviews, with an average rating of 4.68 (out of 5) stars, and 75 are marked as “critical” reviews, with an average rating of 1.77 (out of 5) stars. Meanwhile 75 reviews selected from Amazon China are “positive,” averagely rated 4.54 (out of 5) stars, and the other 75 are “critical,” averagely rated 1.8 (out of 5) stars.

**Table 1:** Amount of online consumer reviews collected in Amazon UK and Amazon China.

Ratings	Amazon UK	Amazon China	Total
Positive	75	75	150
Critical	75	75	150
Total	150	150	300

The reviews have been collected, sorted and ordered according to the popularity. The collecting has been carried out through a web-scraping script written in the Python programming language, which advantageously enables the objectivity of data collection in a highly efficient way, with the dedicated help of a programming professional. In order to guarantee the consistency of research subject and the amount of qualified data, Chinese and English consumer reviews of Kindle Paperwhite are targeted. These reviews are then filtered by their star ratings, which, according to Amazon’s rating rules are developed as follows: five-star and four-star ratings are classified as “positive”; three-star, two-star, and one-star ratings are marked as “critical.” Then the Python script would analyze and include qualified reviews according to the length and location. As the advised length of a review in Amazon is more than 75 words (Virtanen, 2017) and longer reviews usually offer more information and are therefore believed to be more convincing (Pan and Zhang, 2011), only reviews reaching this length range are selected in order to control the quality of review texts. Moreover, to ensure that the reviews collected are written by people who have experienced the good or service, only those reviews of consumers marked as “verified purchase” are selected. The selected reviews are then exported into four excel documents: English Positive Reviews (EPR), English Critical Reviews (ECR), Chinese Positive Reviews (CPR), and Chinese Critical Reviews (CCR). Each document consists of 75 reviews according to the popularity ranking.

### Analytical procedures

With the corpus data collected from Amazon and the coding scheme figured out in *UAM CorpusTool 3.0*, a useful tool for studies in Systemic Functional Linguistics and related fields, the remaining analytical procedures include three major steps.

1. Through coding and annotation, linguistic features appearing in Chinese and English online consumer review texts can be collected, counted and compared. Four files covering all “positive” and “critical” Chinese and English corpus groups are incorporated into the *UAM CorpusTool 3.0*. Attitude resources in Chinese and English texts of online consumer review are then identified and coded. The statistical results of each file are initially collected and counted through the UAM CorpusTool according to their feature coding, and then compared for further analyses. Similarities and differences in the employment of attitude resources, including their frequency and distribution, are clarified.
2. Detailed discourse analysis of the attitude resources used in Chinese and English online consumer reviews is conducted with the theoretical guidance of Martin and White’s (2005) Attitude System. Meanwhile similarities and differences of Interpersonal meaning (Eggins, 2004; Halliday, 2014) constructed through the employment of attitude resources in Chinese and English online consumer reviews are explored.
3. Possible underlying factors that account for the similarities and differences in the employment of attitude resources and the construction of Interpersonal meaning in Chinese and English online reviews are explained, mainly from the perspective of national cultures (Hall, 1976; Hofstede, 2001; Hofstede et al., 2005).

## Results

### The frequency and distribution of attitude resources

Since this study endeavors to contrast specific linguistic features between English and Chinese online reviews, the statistics generated showing the frequency and distribution of attitude resources in both English and Chinese online consumer reviews are presented in Table 2 below. Based on three categories of attitude resources as well as their polarity, similarities and differences in several aspects can be found in the frequency and distribution of attitude resources employed in English and Chinese online consumer reviews.

**Table 2:** Distribution of attitude resources in Chinese and English Amazon consumer reviews.

	Amazon UK		Amazon China	
Features	EPRs (774)	ECRs (535)	CPRs (593)	CCRs (384)
Affect	26.49% (205)	25.98% (139)	20.20% (120)	17.66% (68)
Un/happiness	13.31%(103)	6.54%(35)	4.21%(25)	2.60%(10)
Dis/satisfaction	5.94%(46)	8.22%(44)	8.59%(51)	8.05%(31)
In/security	0.78%(6)	3.36%(18)	2.69%(16)	2.34%(9)
Dis/inclination	6.46%(50)	7.85%(42)	4.71%(28)	4.68%(18)
Judgment	19.90% (154)	24.11% (129)	29.97% (178)	38.96% (150)
Normality	3.62%(28)	10.65%(57)	12.29%(73)	18.18%(70)
Capacity	12.66%(98)	6.73%(36)	10.44%(62)	12.21%(47)
Tenacity	0	0	0	0.26%(1)
Propriety	3.62%(28)	5.42%(29)	7.24%(43)	7.27%(28)
Veracity	0	1.31%(7)	0	1.04%(4)
Appreciation	53.62% (415)	49.91% (267)	49.66% (295)	43.12% (166)
Reaction	24.42%(189)	25.42%(136)	22.90%(136)	16.88%(65)
Composition	23.00% (178)	15.70%(84)	22.39%(133)	17.66%(68)
Valuation	6.20% (48)	8.79%(47)	4.38%(26)	8.57%(33)
Polarity-positive	79.46%(615)	29.72%(159)	56.40%(335)	14.81%(57)
Polarity-negative	20.54%(159)	70.28%(376)	43.43%(258)	84.94%(327)

As Table 2 shows, more attitudinal units are involved in English online consumer reviews than in Chinese ones. To be more specific, a total of 1,309 lexical units that express attitudes can be found in English online consumer reviews while 979 units are found in Chinese reviews. The ratio of attitudinal units between English and Chinese online consumer reviews is also consistent with that of total word count between English and Chinese online consumer reviews.

Another aspect is that the English and Chinese review writers demonstrate a different distribution of affect resources in “positive” reviews and a similar distribution in “critical” reviews. In terms of “positive” reviews, review writers in the United Kingdom employ more lexical units that realize happiness and inclination, while for Chinese review writers relatively more attention is paid to lexical units that realize satisfaction. From the perspective of “critical” reviews, the distribution pattern of affect subcategories is more consistent, with more lexical units conveying dissatisfaction. Another interesting aspect however is that slightly more resources realizing unhappiness and disinclination are employed in ECRs (6.54, 7.85%) than in CCRs (2.60, 4.68%).

There is also a different distribution of judgment resources in the “positive” reviews but a similar distribution in “critical” ones for review writers in the United Kingdom and China. With review writers in both countries employing judgment resources the least among three major categories of attitude resources, review writers in China employ slightly more judgment resources in their online reviews (29.97, 38.96%) than consumers in the United Kingdom (19.90, 24.11%). More capacity resources (12.66%) are used in EPR, while more normality resources are used in CPR. A few resources that indicate tenacity and propriety are involved in Chinese reviews while none exists in those English reviews. Overall, in both countries more judgment resources are employed in “positive” reviews than in “critical” reviews.

Further, review writers in both countries manifest a homogenous distribution of appreciation resources in both “positive” and “critical” reviews. The distribution reveals that among the three subcategories of appreciation resources, lexical units that realize reaction are the most frequently employed (more than 20%) and lexical units that realize valuation are the least employed (less than 10%) in both English and Chinese online consumer reviews. However, compared to review writers in China, review writers in the United Kingdom tend to use slightly more lexical units that realize reaction, while review writers in China pay more attention to lexical units that realize composition.

Finally, consumers from the United Kingdom and China manifest a different distribution of attitude resources in terms of polarity. With more positive resources used in both EPRs and CPRs as well as more negative resources used in both ECRs and CCRs, the overall tendency of polarity in “positive” and “critical” reviews in the two languages is consistent; however, some differences exist. Many negative attitudinal resources (more than 40%) are found in CPRs while most of the attitudinal resources (nearly 80%) in EPRs are found to be positive. In terms of those “critical” reviews, about 85% of the attitudinal units are realized negatively in CCRs while only about 70% are realized negatively in ECRs, and the other 30% are realized positively.

### Attitude resources in English and Chinese online consumer reviews

Under the framework of Martin and White’s Attitude System, attitudinal resources can be sub-divided into three categories: affect, judgment, and appreciation, which indicate different aspects of evaluation. To illustrate how these resources are employed in the reviews, English and Chinese illustrative extracts are presented below, in the original Chinese and with translations included.

#### Affect

The Affect subsystem (Martin and White, 2005) includes lexical units that construe un/happiness, in/security, dis/satisfaction and dis/inclination.

Extract 1: I was drawn to the features available on the All-new Kindle Paperwhite - and I was equally impressed by all the rave reviews (EPR 05).

[+affect: satisfaction]; [+affect: satisfaction].

Extract 1 is taken from a 5-star positive review published in the United Kingdom on November 20, 2018. This review extract follows up the reviewer’s consistent feelings from pre-purchase to post-purchase processes. Synonymous with “be attracted by,” the phrase “be drawn to” is used to express the interest in a person or thing because of their particular quality. Before the purchasing behavior, the reviewer had already become interested in the product—Kindle Paperwhite, for its product features. Meanwhile, referring to the existed reviews of other reviewers who had bought the product, the reviewer was further “impressed,” which indicates that stimulated by those enthusiastic reviews previously published, the reviewer felt greater interest toward the product.

Extract 2: 关注kindle已经有段时间了，新版本发售赶上双十一的活动，终于下定决心入手。(CPR 38).

Guan zhu kindle yi jing you duan shi jian le, xin ban ben fa shou gan shan shuang shi yi de huo dong, zhong yu xia ding jue xin ru shou.

I’d been interested in the kindle for a period of time, and finally *made up my mind* to buy one when the new version was released during the Double Eleven shopping festival.

[+ affect: satisfaction]; [+ affect: inclination].

Extract 2 is selected from a 4-star positive review published in China on November 14, 2018. In the Chinese comment, the review writer has firstly expressed his or her continuous interest in and attention to the product before purchasing, signifying the reviewer’s positive satisfaction toward the product in the pre-purchase period. In describing the impetus for buying brought by the release of a new version and the discounts of Double 11, the reviewer employs the phrase “make up my mind” to imply the change from an emotional intention to actual action.

Extract 3:

*Very disappointing*! I usually *love* Amazon products but I *do not understand* the design of this one. (ECR 38).

[−affect: satisfaction]; [+affect: happiness]; [−affect: security].

Extract 3 is part of a 1-star critical review published in the United Kingdom on February 3, 2019. As the starting part of the review, the adjective “disappointing,” intensified by the adverb “very,” leads the major tone of the whole piece of the review—dissatisfaction with the product. To achieve the expression of dissatisfaction, the reviewer mentions his or her usual affection for the product, which contrasts strongly with the negative feeling experienced with the current purchase. Moreover, the reviewer conveys further insecurity toward the product, showing a questioning attitude and distrust to the product design. In this critical review, a positive affect resource is utilized, but only to contrastively emphasize the overall negative feelings upon receiving the product.

Extract 4:

特别不满意。买过的最不满意的电子产品，没想到是在亚马逊买到的，如果系统可以的话，我一颗星都不想选。(CCR 41).

Te bie bu. man yi. Mai guo de zui bu. man yi de dian zi chan pin, mei xiang dao shi zai ya ma xun mai de, ru guo xi tong ke yi de hua, wo yi ke xing dou bu. xiang xuan.

Extremely disappointed. I did not expect to have bought one of the most disappointing electronic products from Amazon. If the system permitted, I would not like to choose even one star.

[−affect: satisfaction]; [−affect: satisfaction]; [−affect: security]; [−affect: inclination].

Extract 4 is obtained from a 1-star Chinese critical review published on February 3, 2019. The review writer comes straight to the point from the very beginning, informing sellers or potential consumers of a negative attitude toward the purchase by using the phrase “extremely disappointed.” The degree adverb “extremely” intensifies the expression of the reviewer’s dissatisfaction. This purchase is “the most disappointing,” which further indicates the reviewer’s dissatisfaction with this shopping experience compared with previous purchases of electronic products. To more clearly emphasize the dissatisfaction toward the purchase, the review writer employs “did not expect,” which indicates that the quality of this product bought on Amazon falls behind the believed quality of Amazon products in general, and also implies that the reviewer might doubt the quality of products on Amazon in future purchases.

As indicated in Extracts 1–4, consumers tend to employ affect resources to express their mental feelings, which can be positive as well as negative. Positive affect resources are chosen to show consumers’ inner happiness and satisfaction during the whole purchasing experience of certain products or services from the pre-purchase to post-purchase stage. While negative affect resources are mainly employed to reflect dissatisfaction after the purchase, often pointing out poor design or quality, or a disinclination for future purchase. Furthermore, it is noteworthy that some reviewers choose to utilize affect resources with contrastive purposes, such as comparing past positive affection and current dissatisfaction. In such cases, it is always the current feeling of outburst when the review is written that is most prominent.

#### *Judgment*

Judgment, which has something to do with the evaluation of behavior, can be divided into two sub-categories: social esteem and social sanction (Martin and White, 2005).

Extract 5:

After following various procedures and Amazon realizing it wasn’t just a temporary hiccup, I was immediately sent a new one and then returned the faulty one free of charge. (EPR 57).

[+judgement: normality]; [+judgement: capacity]; [+judgement: propriety].

Extract 5 is selected from a 5-star positive review published in the United Kingdom on April 11, 2019. In this short extract, the process of customer service including returning and changing goods is generally described. Initially, Amazon serves in a formal manner by “following” procedures to find the problem. Then it demonstrates its capacity in problem solving by responding quickly and sending a new product to the customer “immediately,” and finally Amazon shows its noble ethical values with its action of returning the faulty goods “free of charge,” which means no costs to the consumer/purchaser. Through the description of a series of behaviors by Amazon in returning and changing goods, the reviewer positively evaluates the capacity as well as the propriety of Amazon.

Extract 6:

很感谢客服主动电话联系说明，因为那个不能用的数据线本来可以给三星，但是客服很尽责，加一星，给四星。(CPR 10).

Hen gan xie ke fu zhu dong dian hua lian xi shuo ming, yin wei na ge bu. neng yong de shu ju xian ben lai ke yi gei san xing, dan shi ke fu hen jin ze, jia yi xing, gei si xing.

Thanks to the customer service for taking the initiative to call and explain. Because of the cable that did not work, a three-star review could have been given, but as the customer service was very responsible, I would add one star and give it four stars.

[+judgement: tenacity]; [+judgement: propriety].

Extract 6 is selected from a 4-star positive review published in China on December 14, 2018. The review writer explains part of the reasons behind his or her rating in this piece of review. With the focus of expressing thanks to the customer service, the writer thinks highly of the tenacity of the customer service worker who reacts proactively to potential conflict. By “taking the initiative,” the customer service successfully controls the situation, which impresses the consumer a lot as an accommodating and reliable person. Moreover, the reviewer continues to highly praise the customer service worker for being “responsible.” Intensified by the degree adverb “very,” the adjective “responsible” is employed as a positive judgment on the propriety of the customer service’s behavior. And because of the customer service’s good moral trait, the review has raised the rating for the goods to four stars, which indicates that the behaviors of other people, especially those who provide relevant services in the purchasing process, can affect consumer ratings.

Extract 7:

I have spent hours speaking to customer service agents on the phone and over chat, each rudely referring me to a new department without asking for permission to do so. (ECR 75).

[−judgement: propriety].

Extract 7 is from a 1-star critical English review on February 27, 2019. The negative judgment erupts due to the impropriety experienced during the purchasing process. This is seen where the reviewer mentions his or her interaction with customer service agents of Amazon: with the evaluative adverb “rudely,” the behavior of those customer service agents is negatively judged in terms of social ethics.

Extract 8:

快递很差，差到我都不想给星级。 (CCR 39).

Kuai di hen cha, cha dao wo dou bu. xiang gei xing ji.

The delivery is poor, and is so poor that I would not like to give one star to it.

[−judgement: capacity]; [−judgement: normality].

Extract 8 is taken from a 1-star critical Chinese review published on November 24, 2018. The reviewer writer targets the delivery in this review, generally evaluating it negatively as it was conducted in a “poor” manner. The adjective is employed repetitively with the first “poor” summarizing the overall negative attitude toward the delivery and the second “poor” emphasizing its low capacity. The second “poor” is employed together with an adverbial clause implying the reviewer’s disinclination to give even one star to the goods, which also represents the reviewer’s negative judgment toward the whole purchasing experience.

To summarize, as Extracts 5–8 indicate, judgment resources can be positively or negatively exploited by review writers to evaluate the behaviors of sellers, customer service workers, delivery staff and anyone else who might engage in the purchasing process. Positive judgment can be made due to the professional and dedicated service of customer service staff, while negative judgment might be given when the product is not properly delivered or packed. Judgment resources can also be applied together with some affect resources such as lexis indicating un/happiness or dis/inclination to strengthen the expression of a certain attitude.

#### *Appreciation*

Appreciation includes three subcategories: reaction, composition and valuation, which can be positive or negative (Martin and White, 2005).

Extract 9:

The kindle paperwhite is definitely worth every penny as it is light to hold, easy to use and waterproof should you take it on your hands. (EPR 75).

[+appreciation: valuation]; [+appreciation: composition];

[+appreciation: composition]; [+appreciation: composition]

Extract 9 is part of a 5-star positive English review published on January 31, 2020. The review writer firmly affirms the value of the product purchased by stating his or her evaluation with lexical units that realize positive appreciation. The verb phrase “worth every penny,” which means the product is great value for buyers, and is employed at the beginning to implicate the reviewer’s positive valuation of the product. The adverb “definitely” is used to emphasize the strength of the positive valuation. In order to support the valuation of product worthiness, the review writer further specifies its basic characteristics or the product features or composition. Three aspects of the product features include “light to hold,” “easy to use” and “waterproof.” The first phrase, “light to hold,” is a positive appreciation of the weight of the product, and the latter two, “easy to use” and “waterproof,” are both positive appreciation of the complexity of the product, implicating the ease of product operation as well as its physical strength.

Extract 10:

机子没啥说的，轻薄、防水、纯平、更均匀的背光，这些特点都挺喜欢，应该是目前最好的kpw了。(CPR 11).

Ji zi mei sha shuo de, qing bao, fang shui, chun ping, geng jun yun de bei guang, zhe xie te dian dou ting xi huan, ying gai shi mu qian zui hao de kpw le.

Nothing to say about the product but its characteristics including the thin, *waterproof*, *flat* and *more uniform* backlight, which I quite like, and it should be *the best* kpw at present.

[+appreciation: composition]; [+appreciation: composition];

[+appreciation: composition]; [+appreciation: reaction]

Extract 10 is obtained from a 5-star positive Chinese review published on November 14, 2018. Similar to the reviewer in Extract 9, the writer of this extract also highlights the characteristics of purchased product. In terms of complexity, the reviewer employs “thin” and “waterproof” to positively appreciate the product, signifying that the product is easy to carry and has advanced design functions. From the aspect of balance, the reviewer evaluates the product by using “flat” and a “more uniform backlight” to present the balance in both the surface and backlight of the product. On account of those great positively appreciated characteristics, the reviewer then summarizes that the product can be considered as one of “the best” products at present, conveying his or her generally positive reaction.

Extract 11:

Really really bad. The design is just flawed to start with! The power button sits at the bottom of the kindle so if you hold it, it switches off. As for the working of the kindle it is shocking, flashing and slow. (ECR 04).

[−appreciation: reaction]; [−appreciation: composition]; [−appreciation: reaction];

[−appreciation: composition]; [−appreciation: reaction]

As part of a 1-star critical review published in the United Kingdom on December 5, 2018, Extract 11 begins with a very negative appreciation of the product, which is intensified by two repeated adverbs “really,” with the adjective “bad” utilized to express the reviewer’s overall negative reaction toward the purchase. The reviewer continues to explain his or her negative appreciation by initially referring to the design which is “flawed,” and the adjective “bad,” meaning a fault in the product design, expresses the reviewer’s negative attitude on its composition. Then the reviewer employs a series of adjectives including “shocking,” “flashing” and “slow” to convey his or her negative reaction toward the working of the product as well as the actual imbalance of light during its operation.

Extract 12:

后壳用的是类肤质材质，观感一般，实际触感很廉价，是大败笔。(CCR 12).

Hou ke yong de shi lei fu zhi cai zhi, guan gan yi ban, shi ji chu gan hen lian jia, shi da bai bi.

The battery cover is made of skin-like material, which has a general appearance. The actual touch is very cheap, which is a big failure. [+appreciation: composition];

[−appreciation: valuation]; [−appreciation: valuation].

Extract 12 is selected from a 2-star critical review published in China on June 23, 2019. In terms of the actual touch or feeling generated from handling the battery cover, the reviewer begins to negatively evaluate it as “very cheap,” which stresses that the battery cover is low in quality, and depreciates it as “a big failure.”

Extracts 9–12 reflect the fact that consumers in both the United Kingdom and China pay great attention to the basic features, quality and worthiness of the products they have purchased. Appreciation resources may include review writers’ instant reaction to the products, the composition of products once they are received and consumers’ valuation toward the products. Furthermore, positive appreciation resources and negative appreciation resources can be combined in a single piece of review.

In summary, evaluations in both English and Chinese online consumer reviews are realized by various attitudinal resources and those attitudinal resources are used to construct Interpersonal meaning, including attitude expression and relationship construction. Resources that realize affect in the online consumer reviews show the review writers’ mental feelings about the design, the content, the purchasing and the delivery of the goods. Judgment resources are used to evaluate the behaviors of the sellers, the delivery staff, the customer service or any people involved in the purchasing process. Appreciation resources represent the review writers’ reactions to, the physical composition of and the social value of the products purchased. Attitudinal resources are differently distributed in English and Chinese online consumer reviews. Applying more attitudinal units, review writers in the United Kingdom tend to use more resources expressing affect in their online reviews, while Chinese consumers employ slightly more judgment resources. Moreover, lots of positive attitudinal resources are found in English “critical” reviews, while many negative attitudinal resources may appear in Chinese “positive” reviews.

## Discussion and conclusion

As the results indicate, Chinese review writers tend to use more judgment resources in online consumer reviews while review writers in the United Kingdom employ more affect resources. Moreover, Chinese review writers tend to use many negative resources in their “positive” reviews while many positive resources are found to be used by consumers from the United Kingdom in their “critical” reviews.

### Cultural dimensions

The major finding of the employment of attitude resources in Chinese and English online consumer reviews echo or align with the respective national cultures. Personal behaviors in particular activities are determined by their national culture (Holliday, 1999), and culture would reflect people’s preferences and attitudes (Mazaheri et al., 2011). Linguistic similarities and differences in the distribution of attitudinal resources in English and Chinese online consumer review can be understood through the lens of variations in national cultures.

#### Indulgence/restraint

One of the major findings suggests that many positive resources are used by consumers from the United Kingdom in their “critical” reviews, and lots of negative resources are employed by Chinese consumers in their published “positive” reviews. People from more indulgent societies tend to enjoy life and be more positive, while people from more restrained societies attach great importance to moral disciplines and tend to be more pessimistic (Hofstede et al., 2010). Similar findings in previous literature (Kim et al., 2018) suggest that people from more indulgent societies such as the United Kingdom tend to enjoy life and be more positive, while people from more restrained societies such as China attach great importance to moral disciplines and tend to be more pessimistic. Thus, consumers from countries with indulgent culture tend to make purchasing decisions in a more spontaneous way and are more likely to give positive review ratings (Kim, 2019).

Since the more indulgent consumers from the United Kingdom are more inclined to enjoy life and to be more positive, they are more likely to be positively predisposed (Kim et al., 2018) and utilize many positive expressions, even in their “critical” online consumer reviews. They focus more on the pursuit of happiness, therefore in their online consumer reviews, more positive attitudinal resources about happiness and satisfaction are employed. For example, in “I feel locked into Amazon and overall I’m happy but yes the ‘special offers’ are still bugging me,” the positive affect resources expressing satisfaction (“feel locked into”) and happiness (“happy”) are overtly and frankly utilized by the review writer in this 3-star critical English review.

By contrast, within a culture of restraint, Chinese consumers are less likely to remember positive emotions and tend to maintain a more pessimistic attitude toward life (Hofstede et al., 2010). As a result, in their “positive” comments, a significant number of negative attitudinal resources are used. A typical example can be shown in “整体使用感受良好，就说不满意的地方吧 Zheng ti shi yong gan shou liang hao, jiu shuo *bu. man yi de* di fang ba” (The overall feeling on the use is good, and here I’d like to talk about what I *am dissatisfied with*). In this 4-star positive review published by Chinese consumers, the review writer starts with an overall positive appreciation toward the whole purchasing experience but then continues with a negative attitude resource that realizes dissatisfaction and indicates a shift to talking about negative aspects in the purchasing in the remainder of the review text, which supports the notion of indulgence and restraint cultures.

#### Individualism/collectivism

Another finding is that more attitude resources are utilized in English online consumer reviews than in Chinese reviews. In those attitude resources, despite the fact that appreciation resources are the most frequently employed, there are more judgment resources in Chinese reviews than writers in the United Kingdom. They employ more affect resources in their reviews. This resonates with previous studies which find that emotional features are stronger and pleasure is the most influential type of emotion in individualistic cultures (Mazaheri et al., 2011).

This dimension of individualism/collectivism is concerned with the relationship between the individual and the group. In more individualistic cultures, personal interests are emphasized and personal freedom is of greater importance, while in more collectivistic cultures, group interests, cooperation and harmonious relationship are held in greater esteem. Being more individualistic, consumers from the United Kingdom are more self-focused, which leads to more attitudinal units in their English reviews, and they attach greater importance to individual affection, bringing more affect resources realizing emotional feelings such as happiness and inclination. Further, within an individualistic culture like the United Kingdom, consumers are less likely to be influenced by external factors in their online reviews (Kim et al., 2018). Therefore, judgment resources, which convey personal attitude towards other people involved in the purchasing experience, are less frequently used in English online consumer reviews.

On the other hand, Chinese consumers, who embrace a more collectivistic culture, are inclined to place the interest of the group before individuals and focus less emphasize on so-called “pleasure” seeking (Mazaheri et al., 2011)., They are therefore less inclined to employ affect resources which convey personal emotions including happiness and inclination. Moreover, consumers from collectivistic cultures tend to be influenced by surrounding, contextual factors in their evaluations of service (Kim et al., 2018), which fosters the view that review writers in China employ more judgment resources and more appreciation resources when composing in their published online reviews.

### High/low-context cultures

In a high-context culture, most of the information would be transmitted in the physical context or internalized in the person with implicit codes so that less information is openly expressed in their language. In a low-context culture, most of the information would be presented in an explicit way by use of the linguistic resources (Hall, 1976; Kim et al., 1998).

Living in a high-context culture, consumers from China use fewer words in their online reviews and apply fewer lexical units to express attitude. And Chinese review writers tend to favor establishing a harmonious and cooperative relationship with potential consumers by providing more comprehensive information. For example, in the employment of judgment resources, lexical units covering normality, capacity, tenacity, propriety and veracity are all employed to better describe the purchasing experience. Also, in the employment of appreciation resources, lexical units about the composition of the product are greatly employed to comprehensively evaluate the complexity and other characteristics about the product. These findings are consistent with previous studies (Hall, 1976; Srivastava and Kalro, 2019) which have identified that members of high-context cultures tend to provide information as a trustworthy reviewer. However, consumers from the United Kingdom demonstrate their low-context culture in review writing by using more words and more attitudinal units in their reviews. Applying more affect resources and appreciation resources on reaction, review writers in the United Kingdom are inclined to place more value on individuality when writing their reviews (Srivastava and Kalro, 2019).

These variations in the dimensions of national cultures account for the differences in the frequency and distribution of attitudinal resources between English and Chinese online consumer reviews. With a more collectivistic, restrained and high-context culture, Chinese consumers are inclined to employ relatively fewer attitudinal resources and tend to apply more judgment and appreciation resources than affect resources. Consumers in the United Kingdom, who embrace a low-context culture encouraging individualism and indulgence, are on the other hand more likely to express personal emotion in their online reviews and therefore more affect resources are utilized.

To sum up, with the aim of investigating Interpersonal meaning of attitude resources employed in Chinese and English online consumer reviews, this study identifies various attitude resources employed in Chinese and English online consumer reviews to express a personal attitude toward or to establish relationship with sellers, customer service, or potential buyers. More attitudinal resources are involved in English Amazon consumer reviews than in Chinese versions. Many positive resources are used by consumers from the United Kingdom in their “critical” reviews, while greater numbers of negative resources are used by Chinese consumers in their “positive” reviews. Factors relating to the similarities and differences in the employment of attitude resources to realize Interpersonal meaning can be clarified by drawing upon a view of national cultures in terms of the notion of high-context and low-context cultures (Hall, 1976; Hofstede, 2001; Hofstede et al., 2005). As a collectivistic and restrained country within a high-context culture, China incorporates different values and thus different choices in language use, compared with the United Kingdom, which is more individualistic and indulgent, where the context is a more low-context culture.

## Data availability statement

The original contributions presented in the study are included in the article/supplementary material, further inquiries can be directed to the corresponding author.

## Author contributions

All authors listed have made a substantial, direct, and intellectual contribution to the work and approved it for publication.

## Funding

This paper is sponsored by General University Innovation Team Project of Guangdong Province (2021WCXTD007).

## Conflict of interest

The authors declare that the research was conducted in the absence of any commercial or financial relationships that could be construed as a potential conflict of interest.

## Publisher’s note

All claims expressed in this article are solely those of the authors and do not necessarily represent those of their affiliated organizations, or those of the publisher, the editors and the reviewers. Any product that may be evaluated in this article, or claim that may be made by its manufacturer, is not guaranteed or endorsed by the publisher.

## Footnotes

^1^ www.amazon.co.uk

^2^ www.amazon.cn
